# Molecular and microbiological report of a hospital outbreak of NDM-1-carrying *Enterobacteriaceae* in Mexico

**DOI:** 10.1371/journal.pone.0179651

**Published:** 2017-06-21

**Authors:** Paola Bocanegra-Ibarias, Elvira Garza-González, Rayo Morfín-Otero, Humberto Barrios, Licet Villarreal-Treviño, Eduardo Rodríguez-Noriega, Ulises Garza-Ramos, Santiago Petersen-Morfin, Jesus Silva-Sanchez

**Affiliations:** 1 Facultad de Ciencias Biológicas, Universidad Autónoma de Nuevo León, San Nicólas de los Garza, Nuevo León, Mexico; 2 Servicio de Gastroenterología, Hospital Universitario Dr. José Eleuterio González, Universidad Autónoma de Nuevo León, Monterrey, Nuevo León, Mexico; 3 Instituto de Patología Infecciosa y Experimental, Centro Universitario de Ciencias de la Salud, Hospital Civil de Guadalajara Fray Antonio Alcalde, Universidad de Guadalajara, Guadalajara, Jalisco, Mexico; 4 Centro de Investigación Sobre Enfermedades Infecciosas, Instituto Nacional de Salud Pública, Cuernavaca, Morelos, Mexico; Leibniz-Institute DSMZ, GERMANY

## Abstract

**Objectives:**

To characterize the microbiological, molecular and epidemiological data of an outbreak of carbapenem-resistant *Enterobacteriaceae* (CRE) in a tertiary-care hospital in Mexico.

**Methods:**

From September 2014 to July 2015, all CRE clinical isolates recovered during an outbreak in the Hospital Civil "Fray Antonio Alcalde" in Jalisco, Mexico were screened for antimicrobial susceptibility, carbapenemase production, carbapenemase-encoding genes, and plasmid profiles. Horizontal transfer of imipenem resistance; and clonal diversity by pulsed-field gel electrophoresis (PFGE) and multilocus sequence typing (MLST); as well as biofilm production and the presence of 14 virulence genes were analyzed in selected isolates.

**Results:**

Fifty-two carbapenem-resistant isolates corresponding to 5 species were detected, i.e., *Klebsiella pneumoniae* (n = 46), *Enterobacter cloacae* (n = 3), *Escherichia coli* (n = 1), *Providencia rettgeri* (n = 1) and *Citrobacter freundii* (n = 1) with carbapenemase encoding genes *bla*_NDM-1_ (n = 48), *bla*_VIM_ (n = 3), *bla*_IMP_ (n = 1) and *bla*_KPC_ (n = 1) detected in these isolates.

The *bla*_NDM-1_ gene was detected in plasmids from 130- to 170-kb in *K*. *pneumoniae* (n = 46); *E*. *cloacae* (n = 3), *E*. *coli* (n = 1) and *P*. *rettgeri* (n = 1). The transfer of plasmids harboring the *bla*_NDM-1_ gene was obtained in eight transconjugants. One plasmid restriction pattern was detected, with the *bla*_NDM-1_ identified in different restriction fragments.

Predominant clone A of *K*. *pneumoniae* isolates archived 28/46 (60%) isolates and belongs to ST392. Besides, ST307, ST309, ST846, ST2399, and ST2400 were detected for *K*. *pneumoniae*; as well as *E*. *cloacae* ST182 and *E*. *coli* ST10.

The *fimA* and *uge* genes were more likely to be identified in *K*. *pneumoniae* carbapenem-susceptible isolates (p = <0.001) and biofilm production was more liable to be observed in carbapenem-resistant isolates (p = <0.05).

**Conclusions:**

Four *Enterobacteriaceae* species harboring the *bla*_NDM-1_ gene were detected in a nosocomial outbreak in Mexico; horizontal transfer and strain transmission were demonstrated for the *bla*_NDM-1_ gene. Given the variation in the size of the plasmid harboring *bla*_NDM-1_, complex rearrangements must also be occurring.

## Introduction

Nosocomial infections caused by carbapenem-resistant *Enterobacteriaceae* (CRE) are of particular concern since they can spread rapidly worldwide, and few treatment options remain available for these diseases [1]. Several carbapenem resistance mechanisms have been described in bacteria, and one of the most important in Gram-negative species is the production of carbapenemase enzymes [1,2]. A high diversity of carbapenemases has been reported in *Enterobacteriaceae*, including the Ambler class A *bla*_KPC_, class B metallo-β-lactamases, *bla*_VIM_, *bla*_IMP_, and *bla*_NDM_, and class D carbapenemase *bla*_OXA-48_ type [3]. The *bla*_NDM-1_ gene is located most frequently on large conjugative plasmids of several incompatibility groups. These plasmids also harbor genes conferring resistance to almost all antibiotics that are used to treat enterobacterial infections [2,3].

In Mexico, the first report of *bla*_NDM-1_ was identified in *Providencia rettgeri* [4], and it was subsequently identified in a *Klebsiella pneumoniae* pediatric isolate [5]. Furthermore, horizontal transfer and clonal dissemination have been reported in this country, in an outbreak caused by *K*. *pneumoniae*, *Escherichia coli* and *Enterobacter cloacae* strains harboring a 101-kb IncFII plasmid carrying the *bla*_NDM-1_ gene [6].

The analysis of sixteen NDM-1-producing enterobacterial isolates from eight countries showed that the spread of the *bla*_NDM-1_ gene is not related to specific clones, specific plasmids, or a single genetic structure [3,7]. The rapid and successful spread of carbapenem-resistant NDM-1-positive organisms may be associated with other antibiotic resistance mechanisms; however, the coexistence of multidrug resistance and virulence mechanisms has also been proposed [8].

Several virulence factors have been described in *K*. *pneumoniae*, including adhesins, capsular serotype, iron-scavenging systems, lipopolysaccharide, and biofilm production [9]. The aim of this study was to characterize the epidemiological, microbiological and molecular data of an outbreak of CRE in a tertiary-care hospital in Mexico.

## Materials and methods

### Hospital setting and recognition of outbreak

This study was performed in the Hospital Civil de Guadalajara “Fray Antonio Alcalde” in Jalisco, Mexico. This hospital is an 899-bed tertiary-care teaching hospital located in Guadalajara, the second largest city in Mexico. This hospital provides care to adult and pediatric patients in 31 wards situated among three connected buildings.

In September 2014, resistance to carbapenem was detected in five clinical isolates of *Enterobacteriaceae* in the Laboratory of Bacteriology. The infection control department was alerted, and from there all isolated *Enterobacteriaceae* between September 2014 and July 2015 were collected for analysis. Before September 2014, we had no carbapenem-resistant isolates.

### Ethics statement

The local ethics committee (Comité de Ética en Investigación del Antiguo Hospital Civil de Guadalajara “Fray Antonio Alcalde,” Jalisco, Mexico) approved this study with reference number 003/16. Informed consent was waived by the Ethics Committee because no intervention was involved and no patients identifying information was included.

### Clinical isolates and patients

*Enterobacteriaceae* species were identified by Matrix-Assisted Laser Desorption Ionization- Time-of-Flight Mass Spectrometry (MALDI-TOF MS) using the Bruker Biotyper (Bruker Daltonics, Germany) as described previously [10].

Drug susceptibility was performed for all isolates using the VITEK automated system and confirmed by the broth microdilution method. Guidelines of the European Committee on Antimicrobial Susceptibility Testing (EUCAST) version 6.0 were used for colistin and tigecycline [11]. Guidelines of the CLSI were used for amikacin, gentamicin, ertapenem, imipenem, meropenem, ceftriaxone, trimethoprim/sulfamethoxazole, aztreonam, ampicillin, ciprofloxacin, fosfomycin, chloramphenicol [12]. Multidrug-Resistance (MDR) was defined as non-susceptibility to one or more agents of at least three different antibiotic classes [13].

Carbapenem-resistant isolates were screened to detect carbapenemase production using the CarbaNP test [12], and to detect the carbapenemase-encoding genes for *bla*_KPC_, MBL (*bla*_VIM_, *bla*_IMP_, and *bla*_NDM_) and *bla*_OXA-48_ by PCR [14–16]. PCR products were sequenced using the chain termination method with a Big-Dye Terminator kit (Applied Biosystems Foster City, CA) and ABI PRISM 3130 (Applied Biosystems). ESBL encoding genes (*bla*_TEM_, *bla*_SHV_, *bla*_CTX-M_ and *bla*_CYM_), *mcr-1* and *mcr-2* genes were screened by PCR [17–19].

We recovered demographic and clinical data from patients infected with a carbapenem resistant-carbapenemase producer isolates. From each enterobacterial species, one isolate per patient was analyzed.

### Plasmid analysis

Plasmid profiles were obtained from all carbapenem-resistant isolates according to the method described by Kieser [20]. In isolates that presented different plasmid profile according to Kieser method, the S1 nuclease assay was performed [21].

Horizontal transfer of carbapenem resistance by bacterial conjugation with *E*. *coli* J53-2 as the recipient strain was performed by liquid and solid-phase mating as described [22,23] in isolates that presented a different plasmid profile according to S1 nuclease assay. Transconjugants were selected on Luria-Bertani (LB) agar supplemented with rifampin (100 μg/ml) plus imipenem (2 μg/ml) when the conjugation was unsuccessful the assay was performed in LB agar supplemented with rifampin (100 μg/ml) plus cefoxitin (30 μg/ml). Enzymatic digestion with HinIII (Invitrogen, California, USA) was performed in transconjugants with only one plasmid present. The incompatibility groups were detected by PCR replicon typing in these isolates [24].

Additionally, Southern hybridization with a non-radioactive probe (ECL direct nucleic acid labeling and detection system; GE Healthcare, Piscataway, NJ) of the *bla*_NDM-1_ gene was performed in transconjugants and *bla*_NDM-1_ positive isolates.

### Clonal diversity studies

Clonal diversity was performed by pulsed-field gel electrophoresis (PFGE) and Multilocus sequence typing (MLST) analysis of selected isolates. For PFGE, chromosomal DNA was prepared using the methodology described by Kaufmann [25] with some modifications. Chromosomal DNA from the isolates was digested with 10 U of XbaI (Takara Bio Inc., Shiga, Japan) with following conditions: temperature of 14°C, the voltage of 6 V/cm, run time of 23 h, and switch time of 1–30 s. PFGE patterns were analyzed visually, and when the restriction patterns presented 100% similarity, the isolates were classified as a clone. When two or three difference in the restriction pattern were detected the isolates were considered as subtypes as suggested by Tenover *et al*. [26].

MLST was performed on selected isolates harboring *bla*_NDM-1_ gene according to species, PFGE pattern and plasmid analysis using the MLST websites: http://bigsdb.pasteur.fr, http://mlst.warwick.ac.uk and http://pubmlst.org [27–29].

### Virulence factors

Detection of genes encoding virulence factors and determination of biofilm formation were conducted only for the *K*. *pneumoniae* isolates. For a comparison of virulence factors of carbapenem-resistant (*bla*_NDM-1_ positive) and carbapenem-susceptible isolates, a group of twenty-three carbapenem-susceptible isolates was randomly selected for analysis. The susceptible isolates were obtained in the same period of the carbapenem-resistant isolates (September 2014-June 2015) and were collected from similar specimens and hospital wards to carbapenem-resistant isolates.

Virulence genes from *K*. *pneumoniae* [serotypes K1 and K2, *rmpA*, *rmpA2* (regulator of mucoid phenotype), *uge* (uridine diphosphate galacturonate-4 epimerase), *ureA* (urease), *entB* (enterobactin), *iroB* (salmochelin), *irp2* (yersiniabactin), *iucA* (aerobactin), *fimA* (fimbrial), *fimH* (fimbrial), *mrkA* (fimbrial), and *mrkD* (fimbrial)] were screened by PCR [30].

Furthermore, semi-quantitative determination of biofilm formation was performed in these isolates (both carbapenem-resistant and carbapenem susceptible) by crystal violet staining as previously described by Bandeira *et al*., with modifications described by Burmølle *et al*. [9,31]. The biofilm index (OD_595_/OD_600_) was used to normalize the amount of biofilm formed to the total cell content of each sample tested. The biofilm production was classified using the biofilm index as non-adherent (<0.90), weakly adherent (>0.90–<1.20) and strongly adherent (>1.20) The cut-off values were defined according to a comparison in the classification with others methodologies previously reported [32]. *Staphylococcus aureus* ATCC 29213 (a high biofilm producer) and *E*. *coli* ATCC 25922 (a low biofilm producer) were used as quality control organisms.

### Statistical analysis

The similarity coefficients were generated from a similarity matrix calculated using the Jaccard’s coefficient.

Percentages of biofilm production and from each virulence factor were compared using Mann-Whitney test. A P value less than 0.05 was considered statistically significant. Statistical analysis was performed in the SPSS Statistics 22 software (IBM Corporation, Somers, NY, USA).

## Results

### Species and resistance genes

During the eleven months of study, 3044 isolates of *Enterobacteriaceae* were recovered; 86/3044 (2.83%) of them were carbapenem-resistant and carbapenemase-producers. From each enterobacterial species, one isolate per patient was selected for the study (52 isolates).

Five species were found to produce a carbapenemase: *K*. *pneumoniae* (n = 46, 88%), *E*. *cloacae* (n = 3, 6%), *E*. *coli*, *P*. *rettgeri* and *Citrobacter freundii* (n = 1, 2% each).

The *bla*_NDM-1_ gene was detected in 48/52 (92.3%) isolates belonging to *K*. *pneumoniae* (n = 43, 90%), *E*. *cloacae* (n = 3, 6%), *E*. *coli* and *P*. *rettgeri* (n = 1, 2%, each).

The *bla*_VIM_ gene was detected in 3/52 (5.6%) isolates belonging to *K*. *pneumoniae* (n = 2) and *C*. *freundii* (n = 1). The *bla*_KPC_ gene was detected in one isolate of *K*. *pneumoniae*, and the *P*. *rettgeri* isolate harbored two carbapenemase genes (*bla*_NDM-1_ and *bla*_IMP_). The *bla*_OXA-48_ gene was not detected in any of the isolates.

**Fig 1:**
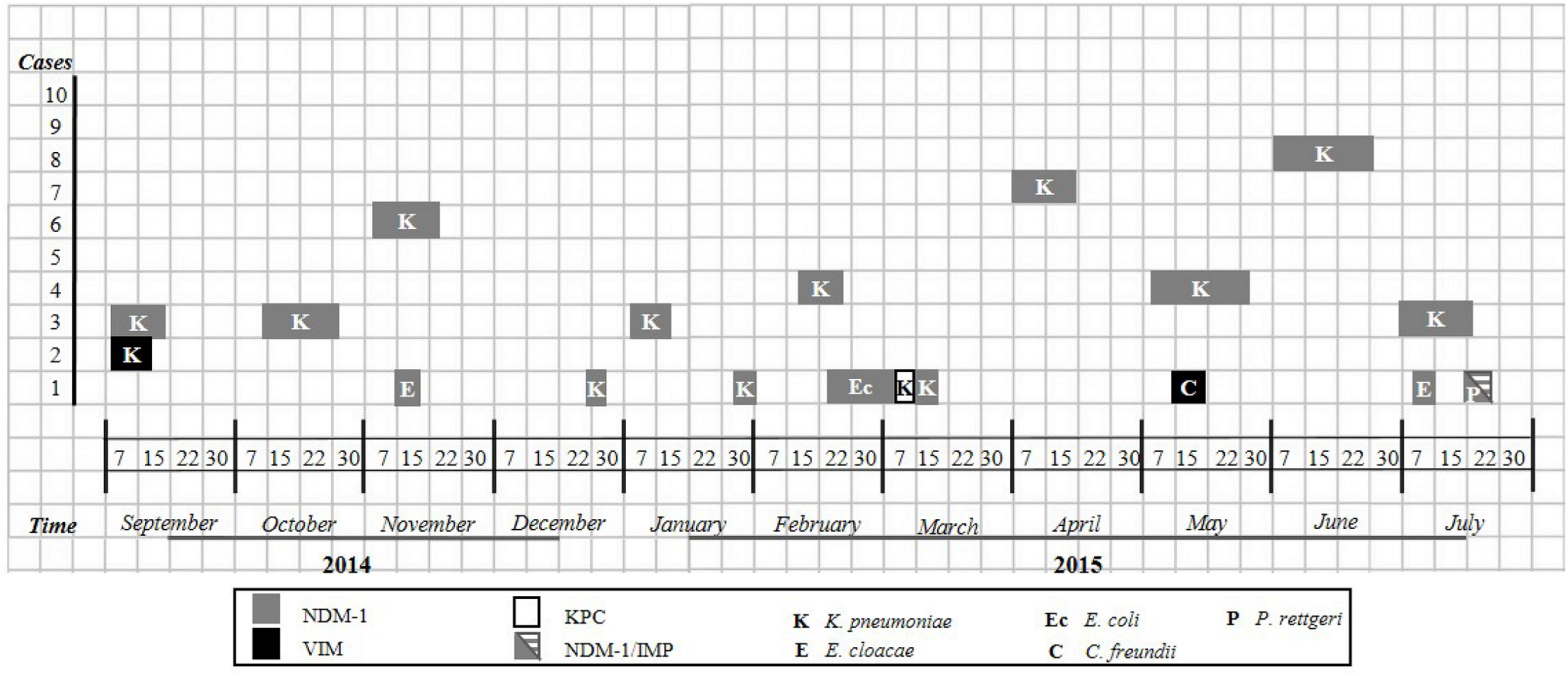
Temporal distribution of recovered carbapenem-resistant *Enterobacteriaceae* isolates from September 2014 to July 2015. Grey squares represent *bla*_NDM-1_; black squares represent *bla*_VIM_; white squares represent *bla*_KPC_; grey and lined squares represent the *bla*_NDM-1_/*bla*_IMP_. K represents *K*. *pneumoniae*; E represents *E*. *cloacae;* Ec represents *E*. *coli;* C represents *C*. *freundii*; and P represents *P*. *rettgeri*.

The first carbapenem-resistance isolate was an NDM-1-producing *K*. *pneumoniae* (Fig 1). Isolates harboring the *bla*_NDM-1_ gene were detected throughout the study.

The *bla*_SHV_ gene was detected in 45/52 (86.5%) isolates, the *bla*_CTX-M_ gene was detected in 39/52 (75%) isolates, the *bla*_TEM_ gene was detected in 30/52 (57.7%) isolates and, *bla*_CYM_, *mcr-1*, and *mcr-2* genes were not detected in any isolate.

### Demographic and clinical data

The 52 isolates were recovered from 51 patients, with one of them was co-infected with two *bla*_NDM-1_ species (*E*. *cloacae* and *E*. *coli*). The mean age was 44 (0–81) years; 66.7% were male, and the mean length of stay (LOS) before positive culture was 26 (range 3–76) days. Clinical characteristics and outcome of the patients are shown in S1 Table. The most frequent cause of hospitalization was brain injury (20% of patients), followed by lower respiratory tract infection (15%), chronic renal failure, and burn injuries (7.5% each).

### Antimicrobial susceptibility patterns

Regarding all the 52 CRE, resistance to ampicillin (100%), ceftriaxone (100%), trimethoprim/sulfamethoxazole (98%), ertapenem (96%), fosfomycin (92%), meropenem (90%), imipenem (88%), ciprofloxacin (87%), gentamicin (83%), amikacin (79%), and aztreonam (73%) was detected. Lower resistance to chloramphenicol (52%), tigecycline (19%), and colistin (4%) was observed. The two colistin-resistant isolates corresponded to *K*. *pneumoniae* positives for *bla*_NDM-1_ and the patients were treated with carbapenems and colistin before the CRE isolation, and after the CRE isolation, a combined therapy of colistin and tigecycline was used.

From the CRE isolates, 51/52 (98%) were classified as MDR and the single non-MDR isolate corresponded to *C*. *freundii* positive for *bla*_VIM_ carbapenemase. The *C*. *freundii* isolate was susceptible to the three carbapenems tested by microdilution but resistant to meropenem and ertapenem using the VITEK system. The other *K*. *pneumoniae* isolates that harbored *bla*_VIM_ presented only resistance to imipenem.

Considering the *E*. *cloacae* isolates, all were resistant to all antibiotics tested except for tigecycline (one strain resistant).

Regarding only the NDM-1-producing *K*. *pneumoniae*, a high drug resistance was detected. The minimum inhibitory concentration (MIC) range, MIC_90_, and MIC_50_, as well as the percentage of resistant and susceptible isolates to each of the antimicrobial agents tested, are indicated in Table 1.

**Table 1:** Antimicrobial susceptibility of *bla*_NDM-1_ producing *K*. *pneumoniae* isolates (n = 43).

Antibiotic	MIC (mg/L)			Isolates n (%)	
	Range	50%	90%	Susceptible	Resistant
Amikacin	≤4–≥ 128	≥ 128	≥ 128	6 (14)	37 (86)
Gentamicin	≤1- ≥ 32	≥ 32	≥ 32	5 (11.6)	38 (88.4)
Ertapenem	4–≥ 128	16	32	0 (0)	43 (100)
Imipenem	2–≥256	8	16	0 (0)	38 (88.4)
Meropenem	2–256	8	16	1 (2.3)	40 (93)
Ceftriaxone	≥ 64	≥ 64	≥ 64	0 (0)	43 (100)
Trimethoprim/ Sulfamethoxazole	8/152–16/304	16/304	16/304	0 (0)	43 (100)
Aztreonam	≤2- ≥128	16	32	7 (16.3)	32 (74.4)
Ampicillin	64- ≥128	≥ 128	≥ 128	0 (0)	43 (100)
Ciprofloxacin	≤0.5–≥16	≥ 16	≥ 16	3 (7)	38 (88.4)
Fosfomycin	256–≥512	512	≥ 512	0 (0)	43 (100)
Chloramphenicol	≤4- ≥128	16	128	6 (14)	20 (46.5)
Colistin	≤0.5- ≥16	≤0.5	2	41 (95.3)	2 (4.7)
Tigecycline	≤0.5–8	2	4	7 (16.3)	9 (21)

Classification of resistance and susceptibility to amikacin, gentamicin, ertapenem, imipenem, meropenem, ceftriaxone, trimethoprim/sulfamethoxazole, aztreonam, ampicillin, ciprofloxacin, fosfomycin, chloramphenicol was based on CLSI interpretive criteria. Classification of resistance and susceptibility to colistin and tigecycline was based on EUCAST interpretative criteria.

### Plasmid pattern, hybridization and transfer of carbapenem resistance

The plasmid profiles of all the 52 CRE clinical isolates harbored between one and five plasmids with sizes of 40 to170 kb. The plasmid profile of NDM-harboring *Enterobacteriaceae* isolates (n = 48) was heterogeneous with 15 different sizes of plasmids according to Kieser method and eight different sizes by S1 nuclease assay.

**Fig 2:**
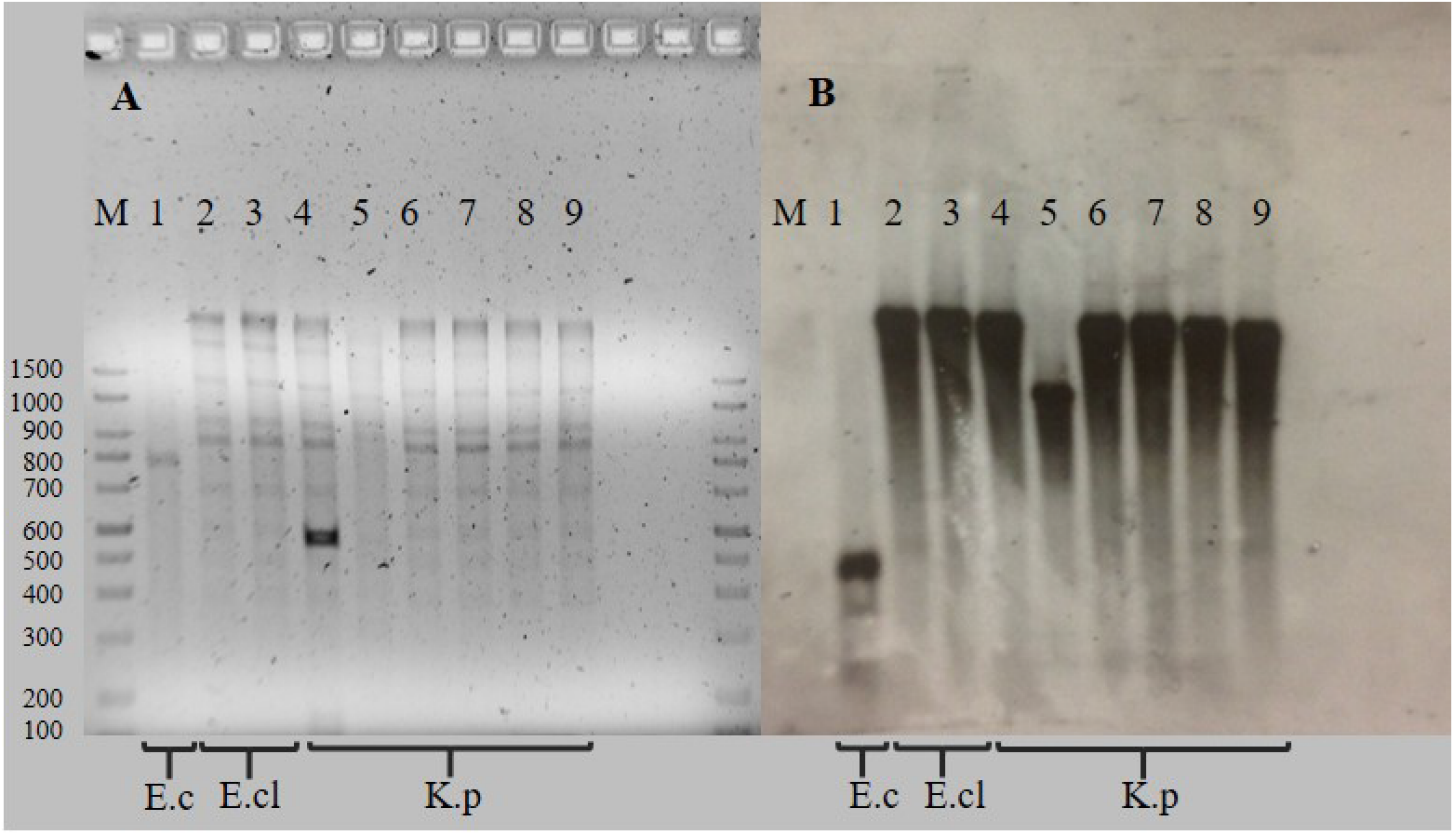
Restriction pattern and Southern hybridization of selected plasmids. (A): Restriction pattern; (B): Southern hybridization; M: Molecular weight marker of 1.5 kb; 1: 15–1327; 2: TT 14–3442 *E*. *cloacae*; 3: TT 15–0026; 4: TT 14–3335; 5: TT 14–3337; 6: TT 14–3424; 7: TT 14–3425; 8: TT 15–1363; 9: TT 15–1372. TT represents transconjugants; E.cl represents *E*. *cloacae;* E.c represents *E*. *coli;* and K.p. represents *K*. *pneumoniae*.

Characteristics of 15 representative isolates of *Enterobacteriaceae* harboring *bla*_NDM-1_ are shown in Table 2. Thirteen isolates (*K*. *pneumoniae*, n = 11; and *E*. *cloacae*, n = 2) were successful in transfer the resistance with eight transconjugants receiving only one plasmid, (from 130 to 150 kb) and contained the *bla*_NDM-1_ gene. Enzymatic digestion revealed one unique restriction pattern, with the *bla*_NDM-1_ gene present on a >1.5 kb fragment in seven transconjugants and a smaller 1.0 Kb fragment in one transconjugant (Fig 2). The IncFIIk and IncFIIy incompatibility groups were identified in the eight transconjugants and the *E*. *coli* isolate that presented a single plasmid, in 88.9% (8/9) and 22.2% (2/9) of the plasmids, respectively.

**Table 2:** Characteristics of representative isolates of *Enterobacteriaceae* harboring *bla*_NDM-1_.

**Clinical isolate data**															
Strain ID	14–3335	14–3337	14–3338	14–3423	14–3424	14–3425	14–3442	15–0026*	15–1880	15–1327*	15–1363	15–1362	15–1372	15–1887	15–1941
Species	*K*. *pn*	*K*. *pn*	*K*. *pn*	*K*. *pn*	*K*. *pn*	*K*. *pn*	*E*. *cl*	*E*. *cl*	*K*. *pn*	*E*. *coli*	*K*. *pn*	*K*. *pn*	*K*. *pn*	*K*. *pn*	*P*. *re*
**Susceptibility. MIC mg/L (Interpretation)**															
AMP	>128	64	>128	>128	>128	>128	>128	>128	>128	>128	>128	>128	>128	>128	>128
CRO	>64	>64	>64	>64	>64	>64	>64	>64	>64	>64	>64	>64	>64	>64	>64
ETP	>128	4	8	16	32	16	32	128	32	8	32	32	16	16	4
IMP	256	8	8	8	8	8	8	32	256	4	8	4	8	8	4
MEM	256	2	4	8	16	8	16	16	128	4	16	8	4	8	8
ATM	16	<2	8	16	16	16	16	16	16	8	16	<2	16	16	<2
SXT	16/304	16/304	16/304	16/304	16/304	16/304	16/304	16/304	16/304	16/304	16/304	16/304	16/304	16/304	16/304
GEN	>32	<1	16	>32	>32	>32	>32	>32	>32	>32	>32	<1	>32	>32	<1
AMK	>128	<4	16	>128	>128	>128	>128	>128	128	8	>128	16	>128	>128	16
CIP	>16	<0.5	2	>16	>16	>16	>16	>16	>16	2	>16	8	>16	>16	16
FOF	>512	512	256	512	512	512	512	>512	>512	32	>512	256	512	>512	128
CHL	>128	<4	16	32	16	16	>128	>128	32	>128	128	64	32	32	128
TGC	2	<0.5	1	2	2	2	1	<0.5	<0.5	<0.5	2	2	2	8	1
CST	<0.5	<0.5	<0.5	<0.5	<0.5	<0.5	<0.5	<0.5	<0.5	<0.5	<0.5	2	<0.5	<0.5	>16
**Molecular characterization**															
Clone	A (n = 28)	D (n = 1)	E (n = 1)	A1 (n = 2)	A2 (n = 1)	A (n = 28)	A1 (n = 1)	A (n = 2)	A5 (n = 1)	N/A	A3 (n = 2)	G (n = 1)	A4 (n = 1)	A6 (n = 1)	N/A
ST	392	309	846	307	N/D	392	182	N/D	N/D	10	N/D	2400	N/D	N/D	N/A
Plasmid profile by Kieser (No. of isolates)	130, 160, 165 (21)	130, 145, 155 (1)	130, 145, 155 (1)	130, 150, 160, 170 (1)	130, 162, 166 (3)	130, 164, 166 (7)	135, 142, 150 (1)	130, 142, 150 (2)	130, 162, 166 (1)	140 (1)	70, 78, 130, 142, 166 (2)	160, 170 (4)	130,163, 166 (1)	130, 163, 166 (1)	128, 165 (1)
Plasmid profile by S1 nuclease assay	82	82, 90, 96	52, 77, 90, 96	82	82, 90, 96	82	82, 86, 117, 122	82, 100, 117, 142	82	77	82, 102, 133, 142	82	82	82	59
Plasmid carrying NDM-1 (kb)	130	130	130	130, 150	130	130	150	150	130	130	130	170	130	130	-
Positive conjugation (presented a single plasmid)	Yes (Yes)	Yes (Yes)	Yes (No)	Yes (No)	Yes (Yes)	Yes (Yes)	Yes (Yes)	Yes (Yes)	Yes (No)	No	Yes (Yes)	Yes (No)	Yes (Yes)	Yes (No)	No
Incompatibility groups	IIIk	FIIy	N/D	N/D	FIIy	FIIy	FIIy	FIIy	N/D	FIIy, FIIk	FIIy	N/D	FIIy	N/D	No

(*): same patient

K. pn: *K*. *pneumoniae*; E. cl: *E*. *cloacae*; N/D: Not detected; N/A: Not applicable; AMP: ampicillin; CRO: ceftriaxone; ETP: ertapenem; IMP: imipenem; MEM: meropenem; ATM: aztreonam; SXT: trimethoprim/sulfamethoxazole; GEN: gentamicin; AMK: amikacin; CIP: ciprofloxacin; FOF: fosfomycin; CHL: chloramphenicol; TGC: tigecycline; CST: colistin.

The Southern hybridization experiments showed the presence of the *bla*_NDM-1_ gene in four different plasmids, with sizes from 130 to 170 kb. One *bla*_NDM-1_ gene copy was identified in two different plasmids (130 and 150 kb) harbored in *K*. *pneumoniae* (14–3423) isolate. Conjugation was unsuccessful for the *E*. *coli*, and *P*. *rettgeri* clinical isolates. The Southern hybridization experiment was unsuccessful for the *P*. *rettgeri* isolate.

### Clonal diversity

*K*. *pneumoniae* (n = 46) and *E*. *cloacae* (n = 3) isolates were subjected to PFGE assays. Regarding *K*. *pneumoniae*, 14 distinct patterns were detected. The percentage of similarity ranged from 75% to 100%, with restriction patterns of 15–20 bands and the 60.9% (28/46) of the isolates corresponding to clone A; 4.3% (2/46) to clones B and C; and 2.2% (1/46) to clones D to H.

Clone A harbored *bla*_NDM-1_, Clone B, and F strains harbored *bla*_VIM_ and *bla*_KPC_ genes, respectively. The clone A presented six subtypes (19.5%, 9/46) with two or three differences in the restriction pattern in comparison with clone A restriction pattern.

Regarding *E*. *cloacae* isolates, they were classified as closely related with only two different bands in the restriction pattern and are considered as subtypes. The Clone A and A1 presented one and two isolates, respectively.

Regarding MLST assays, selected isolates were *K*. *pneumoniae* (n = 6), *E*. *coli* and E. *cloacae* (n = 1 each). Regarding *K*. *pneumoniae*, only isolates harboring *bla*_NDM-1_ gene and with different plasmid profile were selected. Four ST previously reported were detected (ST392, strain 14–3335; ST309, strain 14–3337; ST846, strain 14–3338; and ST307, strain 14–3423). Furthermore, two new ST were identified (ST2400, strain 15–1362; and ST2399, strain 15–1600). The clone A corresponded to ST392 and was detected during the 11 months of surveillance.

*E*. *coli* (15–1327) isolate corresponded to ST10, and *E*. *cloacae* (14–3442) isolate corresponded to ST182.

### Virulence factors

Virulence factors analyzed in both carbapenem-resistant and carbapenem susceptible isolates showed that the presence of *fimA* and *uge* genes was more likely to be detected in carbapenem-susceptible isolates (P = <0.001) (Table 3).

**Table 3:** Distribution of virulence factors in *bla*_NDM-1_ producing *K*. *pneumoniae* isolates and carbapenem susceptible isolates.

**Group**	Virulence genes										Biofilm production		
	*entB*	*iroB*, K2	*irp2*	*fimA*	*fimH*	*mrkA*	*mrkD*	*uge*	*ureA*	*rmpA*	non-adherent	weakly adherent	strongly adherent
**CR**	97.7% (42/43)	0% (0/43)	60.5% (26/43)	18.6% (8/43)	100% (43/43)	74.4% (32/43)	0% (0/43)	27.9% (12/43)	76.7% (33/43)	0% (0/43)	69.8% (30/43)	4.6% (2/43)	25.6% (11/43)
**CS**	100% (23/23)	13% (3/23)	47.8% (11/23)	65.2% (15/23)	95.7% (22/23)	100% (23/23)	4.3% (1/23)	82.6% (19/23)	100% (23/23)	8.7% (2/23)	100% (23/23)	0% (0/23)	0% (0/23)
**P-value**	NS	NS	0.467	<0.001	NS	NS	NS	<0.001	NS	NS	NS	NS	NS

CR: Carbapenem-resistant isolates; CS: Carbapenem-susceptible isolates. NS: Not significant. *iucA*, *K1*, and *rmpA2* genes were not detected.

Regarding biofilm production, the 81.8% (54/66) of the isolates were classified as non-adherent, 1.5% (1/66) as weakly adherent, and 16.7% (11/66) as strongly adherent. All strongly and weakly adherent isolates were carbapenem-resistant (Table 3). Biofilm production (strong plus weak) was found to be associated with carbapenem resistance (P = <0.05).

## Discussion

In this study, we characterized the epidemiological, microbiological, and molecular data of an outbreak of CRE in a tertiary-care hospital in western Mexico and detected that the most commonly gene identified was *bla*_NDM-1_ in a predominant clone A of *K*. *pneumoniae*. The presence of only one carbapenemase type (*bla*_NDM-1_) involving *K*. *pneumoniae*, *E*. *cloacae*, and *E*. *coli* in four epidemiologically related patients was reported in a tertiary care hospital in Mexico City in 2015 [6], but not comprising the high number of species and isolates reported in this study.

In addition to being detected in *K*. *pneumoniae*, *E*. *cloacae*, and *E*. *coli*, the *bla*_NDM-1_ gene has been reported in *P*. *rettgeri* isolates [4]. In this species, resistance to carbapenems is rarely described, and when is reported, it is mainly associated with *bla*_NDM-1_ [4,33]. In this study, the *P*. *rettgeri* isolate presented both *bla*_NDM-1_ and the *bla*_IMP_ gene. The presence of *bla*_IMP_ in *P*. *rettgeri* has been reported only in Japan [34,35], and to the best of our knowledge, the presence of *bla*_NDM-1_ and *bla*_IMP_ in the same isolate has not been reported to date. Unfortunately, the *P*. *rettgeri* isolate lost the plasmid carrying *bla*_NDM-1_ during experiments, and we were unable to characterize it. Due to the presence of the *bla*_IMP_ gene, the isolate remained carbapenem-resistant.

Of the several carbapenemases previously described worldwide, the *bla*_KPC_ has been reported as the predominant carbapenemase gene associated with CRE intrahospital infections [36]. Nevertheless, in the recent years, *bla*_NDM-1_ gene has frequently been associated with outbreaks, particularly with strains of *K*. *pneumoniae* and *E*. *coli* [3].

We detected more than one CRE species and multiple carbapenemases genes in the same hospital in the same period. This diversity has been previously reported, but in countries geographically distant from Mexico (China and Kuwait) [37,38]. Because of this, our report underscores the importance of active surveillance in all enterobacterial species.

The transfer of plasmids of 130 to170 kb carrying *bla*_NDM-1_ was demonstrated for *K*. *pneumoniae*, and *E*. *cloacae* and these experiments partially explain, the high dissemination inter and intra-species observed during the outbreak. In contrast, the inability of conjugation of *E*. *coli* and *P*. *rettgeri* could explain the lack of dissemination of these species/plasmids during the outbreak.

Similar restriction patterns were detected in eight plasmids of similar size (130 to150 kb) with the *bla*_NDM-1_ gene present on the same restriction fragment in 7/8 plasmids. These results and the different size of plasmids, strongly suggest rearrangements of plasmids during the short period of this outbreak. Rearrangements of plasmids have been previously reported for plasmids harboring *bla*_VIM_ gene [39], but to our knowledge, rearrangements in plasmids encoding *bla*_NDM-1 _have not been previously described.

One of the most significant findings of our study was the high attributable mortality detected for any CRE (35%, 14/40), even that this percentage was lower than previous reports (57.4%, 54/94) in clinical isolates of *K*. *pneumoniae*, *E*. *coli*, *E*. *cloacae*, *C*. *freundii*, *Enterobacter aerogenes*, *Klebsiella oxytoca*, *Raoultella ornithinolytica* and *Raoultella planticola* obtained from 2010 to 2014 in China [37]. Furthermore, a high attributable mortality related to CRE *bla*_NDM-1_ producers was observed, regarding that lower values have been reported (28.6%, 6/21) in Kuwait for *K*. *pneumoniae*, *E*. *coli*, *E*. *cloacae*, *M*. *morganii* and *P*. *stuartii* recovered in 2014 [38].

In our study, the 19.6% isolates were resistant to tigecycline. The high resistance detected is a point of concern because the SENTRY Antimicrobial Surveillance Program reported in 2016 that only 2.6% of CRE from Latin America presented tigecycline resistance [40], and by these new findings, we may infer that tigecycline resistance is increasing.

Colistin resistance was also detected (4%), and this is now a serious global menace as previous reports from India and UK showed percentages of resistance of 6% and 11%, respectively [41]. This study is the second report of colistin-resistant *Enterobacteriaceae* in Mexico [42]. The molecular mechanism of resistance to colistin presented in these colistin-resistant isolates was not acquired due to *mcr* gene was not detected, and additional analysis is ongoing in our group to evaluate the mechanism(s) involved.

We detected *K*. *pneumoniae* ST307, ST392, and ST846 which have been reported as harboring *bla*_KPC,_ *bla*_OXA-48_ and *bla*_NDM-1_ genes [43–45]. In contrast, the *K*. *pneumoniae* ST309 detected has not been related to carbapenemases genes.

*E*. *cloacae* ST182 harboring *bla*_NDM-1_ has been reported in Mexico and Finland [6,46]; the ST10 of *E*. *coli* has only been reported in isolates harboring ESBL genes and not carbapenemase genes [47].

The combination of clonal expansion and horizontal gene transfer demonstrated in this study has been described in Mexico, UK, and Chennai, India [6,41]. In contrast, isolates from Haryana, India, showed an apparent clonal expansion by the demonstration of two types of predominant plasmids [41].

In our study, we evaluated 14 virulence genes in all carbapenem-resistant and in selected carbapenem-susceptible *K*. *pneumoniae* isolates, and we found that the frequency of virulence genes was similar to reported when 6 *K pneumoniae* KPC +, ST258 were analyzed [30].

The virulence gene distribution between both groups was similar except for the *fimA* and *uge* genes. The presence of *fimA* and *uge* genes was more frequently detected in carbapenem-susceptible *K*. *pneumoniae* isolates (P <0.001). The type 1 fimbriae, encoded by *fimA*, contribute to the invasion of bladder cells and biofilm formation. Despite the low frequency of the *fimA* gene in the carbapenem-resistant isolates, these strains were capable of high biofilm production.

In this study, we confirmed that biofilm production is higher in the carbapenem-resistant isolates than in the carbapenem-susceptible isolates as previously reported [8], and the presence of biofilm may contribute to drug resistance. However, more studies are required to define the impact of virulence genes.

This studies had several limitations. First, there was a lack of information about the travel of patients or healthcare workers to places where NDM-1-producing strains are endemic. However, *bla*_NDM-1_ strains been reported in Mexico previously, and the dissemination could be the result of regional transmission. Another significant limitation of our study is the absence of analysis of CRE carriers in the hospital. The above limitations could be resolved in future studies of CRE in this region.

The results obtained in this study indicate that *bla*_NDM-1_ was disseminated horizontally among different species in a tertiary care Hospital in Mexico, also with proof of strain spread predominantly of *K*. *pneumoniae* ST392. We have provided evidence of plasmid transfer but, given the variation in plasmid sizes, complex rearrangements must also be occurring. In this analysis, the presence of other carbapenemase genes encoding *bla*_VIM_, *bla*_KPC_ and *bla*_IMP_ were sporadic.

## Supporting information

S1 Table Clinical characteristics and outcome of patients. (DOC)

S2 Table MICs for *bla*_NDM-1_ transconjugants. (DOC)
