# Supplementary material for: Molecular and microbiological report of a hospital outbreak of NDM-1-carrying Enterobacteriaceae in Mexico
Source: PLoS One. 2017 Jun 21;12(6):e0179651. doi: 10.1371/journal.pone.0179651 (PMC5479539; doi:10.1371/journal.pone.0179651)
Supplement: S1 Table — (DOC) [file pone.0179651.s001.doc]

**S1 Table.** Clinical characteristics and outcome of patients

| **Characteristic** | **No. (% of patients or range)** | | |
| --- | --- | --- | --- |
|  | **CRE**  **(n=51)** | **NDM-1**  **(n=48)** | **Clone A**  **(n=28)** |
| Specimens |  |  |  |
| Urine | 15 (29.4) | 14 (29) | 9 (32.1) |
| Respiratory sources | 11 (21.6) | 9 (18.8) | 7 (25) |
| Secretions | 11 (21.6) | 11 (23) | 5 (17.9) |
| Blood | 8 (15.7) | 7 (14.6) | 3 (10.7) |
| Vascular catheter | 4 (7.8) | 4 (8.3) | 3 (10.7) |
| Peritoneal fluid | 2 (3.9) | 2 (4.2) | 1 (3.6) |
| Hospital wards |  |  |  |
| Internal medicine | 30 (58.8) | 27 (56.3) | 16 (57.1) |
| Neurosurgery | 7 (13.7) | 9 (18.8) | 6 (21.4) |
| Surgery | 7 (13.7) | 6 (12.5) | 3 (10.7) |
| ICU | 5 (9.8) | 3 (6.2) | 3 (10.7) |
| Pediatric | 2 (3.9) | 2 (4.2) | 0 (0) |
| Comorbidities |  |  |  |
| Hypertension | 21 (41.2) | 17 (35.4) | 11 (39.3) |
| Diabetes mellitus | 13 (25.5) | 11 (23) | 8 (28.6) |
| Acute kidney injury | 12 (23.5) | 11 (23) | 6 (21.4) |
| Risk factors |  |  |  |
| Urinary catheterization | 43 (84.3) | 39 (81.2) | 27 (96.4) |
| Mechanical ventilation | 32 (62.7) | 29 (60.4) | 19 (67.9) |
| Central venous catheter | 32 (62.7) | 29 (60.4) | 17 (60.7) |
| Previous surgery | 31 (60.8) | 27 (56.3) | 16 (57.1) |
| ICU stay | 26 (51) | 23 (47.9) | 13 (46.4) |
| Mean LOS before positive culture (range) | 26 (3-76) | 26 (3-76) | 24 (3-62) |
| Corticosteroids use | 11 (21.6) | 11 (23) | 9 (32) |
| Mortality rate  Mortality rate attributable to infection | 35.3%  27.5% | 35.4%  27.1% | 39.3%  32.1% |
| Number of antibiotics used before isolation |  |  |  |
| 0–4 | 21 (41.2) | 17 (35.4) | 9 (32.1)) |
| 5 or more | 30 (58.8) | 31(64.6) | 19 (67.9) |
| Class of antibiotic used before isolation |  |  |  |
| Carbapenems | 38 (74.5) | 35 (72.9) | 18 (64.3) |
| Cephalosporins | 34 (66.7) | 32 (66.7) | 22 (78.6) |
| Linezolid | 27 (53) | 21 (43.8) | 13 (46.4) |
| Tigecycline | 23 (45.1) | 22 (45.8) | 15 (53.6) |
| Rifampicin | 15 (29.4) | 14 (29.2) | 8 (28.6) |
| Colistin | 15 (29.4) | 14 (29.2) | 11 (39.3) |
| Piperacillin/tazobactam | 14 (27.5) | 14 (29.2) | 9 (32.1) |

ICU: intensive care unit; LOS: length of stay. CRE: Carbapenem-resistant *Enterobacteriaceae*
