# Supplementary material for: Molecular and microbiological report of a hospital outbreak of NDM-1-carrying Enterobacteriaceae in Mexico
Source: PLoS One. 2017 Jun 21;12(6):e0179651. doi: 10.1371/journal.pone.0179651 (PMC5479539; doi:10.1371/journal.pone.0179651)
Supplement: S2 Table — (DOC) [file pone.0179651.s002.doc]

**S2 Table. MICs for *bla*_NDM-1_ transconjugants.**

| **Antibiotic** | **J53-2** | **14-3335** | **Tc 14-3335** | **14-3337** | **Tc 14-3337** | **14-3338** | **Tc 14-3338** | **14-3423** | **Tc 14-3423** | **14-3424** | **Tc 14-3424** | **14-3425** | **Tc 14-3425** | **14-3442** | **Tc 14-3442** | **15-0026** | **Tc 15-0026** | **15-1880** | **Tc 15-1880** | **15-1363** | **Tc 15-1363** | **15-1362** | **Tc 15-1362** | **15-1372** | **Tc 15-1372** | **15-1887** | **Tc 15-1887** |
| --- | --- | --- | --- | --- | --- | --- | --- | --- | --- | --- | --- | --- | --- | --- | --- | --- | --- | --- | --- | --- | --- | --- | --- | --- | --- | --- | --- |
| AMP | 16 | >128 | >128 | 64 | >128 | >128 | >128 | >128 | >128 | >128 | >128 | >128 | >128 | >128 | >128 | >128 | >128 | >128 | >128 | >128 | >128 | >128 | >128 | >128 | >128 | >128 | >128 |
| CRO | <4 | >64 | >64 | >64 | >64 | >64 | >64 | >64 | >64 | >64 | >64 | >64 | >64 | >64 | >64 | >64 | >64 | >64 | >64 | >64 | >64 | >64 | >64 | >64 | >64 | >64 | >64 |
| ETP | <0.5 | >128 | 16 | 4 | 16 | 8 | 16 | 16 | 32 | 32 | 16 | 16 | 16 | 32 | 16 | 128 | 16 | 32 | 32 | 32 | 16 | 32 | 8 | 16 | 16 | 16 | 16 |
| IMP | <1 | 256 | 8 | 8 | 4 | 8 | 4 | 8 | 8 | 8 | 4 | 8 | 4 | 8 | 8 | 32 | 8 | 256 | 8 | 8 | 8 | 4 | 8 | 8 | 8 | 8 | 16 |
| MEM | <1 | 256 | 16 | 2 | 8 | 4 | 8 | 8 | 16 | 16 | 16 | 8 | 16 | 16 | 16 | 16 | 16 | 128 | 16 | 16 | 16 | 8 | 8 | 4 | 16 | 8 | 8 |
| ATM | <2 | 16 | <2 | <2 | 32 | 8 | 32 | 16 | <2 | 16 | <2 | 16 | <2 | 16 | <2 | 16 | <2 | 16 | <2 | 16 | <2 | <2 | <2 | 16 | <2 | 16 | <2 |
| SXT | <0.5/9.5 | 16/304 | <0.5/9.5 | 16/304 | <0.5/9.5 | 16/304 | 16/304 | 16/304 | <0.5/9.5 | 16/304 | <0.5/9.5 | 16/304 | <0.5/9.5 | 16/304 | <0.5/9.5 | 16/304 | <0.5/9.5 | 16/304 | <0.5/9.5 | 16/304 | <0.5/9.5 | 16/304 | 16/304 | 16/304 | <0.5/9.5 | 16/304 | <0.5/9.5 |
| GEN | <1 | >32 | >32 | <1 | 32 | 16 | 32 | >32 | >32 | >32 | >32 | >32 | >32 | >32 | >32 | >32 | >32 | >32 | >32 | >32 | >32 | <1 | <1 | >32 | >32 | >32 | >32 |
| AMK | <4 | >128 | >128 | <4 | 16 | 16 | 8 | >128 | >128 | >128 | >128 | >128 | >128 | >128 | >128 | >128 | >128 | 128 | >128 | >128 | >128 | 16 | >128 | >128 | >128 | >128 | >128 |
| CIP | <0.5 | >16 | <0.5 | <0.5 | 1 | 2 | 2 | >16 | <0.5 | >16 | <0.5 | >16 | <0.5 | >16 | <0.5 | >16 | <0.5 | >16 | <0.5 | >16 | <0.5 | 8 | <0.5 | >16 | <0.5 | >16 | <0.5 |
| FOF | <16 | >512 | <16 | 512 | <16 | 256 | <16 | 512 | 32 | 512 | <16 | 512 | <16 | 512 | <16 | >512 | <16 | >512 | <16 | >512 | <16 | 256 | <16 | 512 | 64 | >512 | 16 |
| CHL | <4 | >128 | <4 | <4 | <4 | 16 | <4 | 32 | <4 | 16 | <4 | 16 | <4 | >128 | <4 | >128 | <4 | 32 | <4 | 128 | <4 | 64 | 8 | 32 | <4 | 32 | <4 |
| TGC | <0.5 | 2 | <0.5 | <0.5 | <0.5 | 1 | <0.5 | 2 | <0.5 | 2 | <0.5 | 2 | <0.5 | 1 | <0.5 | <0.5 | <0.5 | <0.5 | <0.5 | 2 | <0.5 | 2 | <0.5 | 2 | <0.5 | 8 | <0.5 |
| CST | <0.5 | <0.5 | <0.5 | <0.5 | <0.5 | <0.5 | <0.5 | <0.5 | <0.5 | <0.5 | <0.5 | <0.5 | <0.5 | <0.5 | <0.5 | <0.5 | <0.5 | <0.5 | <0.5 | <0.5 | <0.5 | 2 | <0.5 | <0.5 | <0.5 | <0.5 | <0.5 |

Tc: Transconjugant; AMP: ampicillin; CRO: ceftriaxone; ETP: ertapenem; IMP: imipenem; MEM: meropenem; ATM: aztreonam; SXT: trimethoprim/sulfamethoxazole; GEN: gentamicin; AMK: amikacin; CIP: ciprofloxacin; FOF: fosfomycin; CHL: chloramphenicol; TGC: tigecycline; CST: colistin.
